# The SQUIRE (Standards for QUality Improvement Reporting Excellence) guidelines for quality improvement reporting: explanation and elaboration

**DOI:** 10.1136/qshc.2008.029058

**Published:** 2008-09-26

**Authors:** G Ogrinc, S E Mooney, C Estrada, T Foster, D Goldmann, L W Hall, M M Huizinga, S K Liu, P Mills, J Neily, W Nelson, P J Pronovost, L Provost, L V Rubenstein, T Speroff, M Splaine, R Thomson, A M Tomolo, B Watts

**Affiliations:** 1 Quality Literature Program, The Dartmouth Institute for Health Policy and Clinical Practice; Office of Research and Innovation in Medical Education, Dartmouth Medical School, Hanover, New Hampshire, and White River Junction VA Hospital, White River Junction, Vermont, USA; 2 Alice Peck Day Memorial Hospital, Lebanon, New Hampshire, USA; 3 General Internal Medicine; VA National Quality Scholars Program, Birmingham VA Medical Center, University of Alabama at Birmingham, Birmingham, Alabama, USA; 4 Dartmouth Hitchcock Leadership and Preventive Medicine Residency, Obstetrics and Gynecology and Community and Family Medicine, Dartmouth Hitchcock Medical Center, Lebanon, New Hampshire, USA; 5 Institute for Healthcare Improvement (IHI), Harvard Medical School, Boston, Massachusetts, USA; 6 University of Missouri Health Care, School of Medicine, University of Missouri-Columbia, Columbia, Missouri, USA; 7 General Internal Medicine, Vanderbilt University Medical Center, Nashville, Tennessee, USA; 8 Departments of Medicine and Community and Family Medicine, Dartmouth Medical School, Hanover, New Hampshire, USA; 9 VA National Center for Patient Safety Field Office, White River Junction, Vermont, and Dartmouth Medical School, Hanover, New Hampshire, USA; 10 Field Office of VHA’s National Center for Patient Safety, White River Junction, Vermont, USA; 11 Rural Ethics Initiatives, Dartmouth Institute for Health Policy and Clinical Practice, Dartmouth Medical School, Hanover, New Hampshire, USA; 12 Departments of Anesthesiology and Critical Care, Surgery, and Health Policy and Management, Center for Innovations in Quality Patient Care, Quality and Safety Research Group, The Johns Hopkins University School of Medicine, Baltimore, Maryland, USA; 13 Associates in Process Improvement, Austin, Texas, USA; 14 VA Greater Los Angeles and University of California Los Angeles, VA HSRD Center of Excellence for the Study of Healthcare Provider Behavior, RAND, VA Greater Los Angeles at Sepulveda, North Hills, California, USA; 15 Vanderbilt University School of Medicine, VA Tennessee Valley Healthcare System, Nashville, Tennessee, USA; 16 VA National Quality Scholars Fellowship Program, The Dartmouth Institute for Health Policy and Clinical Practice, Dartmouth Medical School, Hanover, New Hampshire, USA; 17 Institute of Health and Society Medical School, Framlington Place, Newcastle upon Tyne, UK; 18 Emergency Department, Louis Stokes Cleveland DVAMC, Case Western Reserve University SOM, Cleveland, Ohio, USA; 19 Inpatient Quality Improvement, Department of Medicine, Louis Stokes Cleveland VA Medical Center, Cleveland, Ohio, USA

## Abstract

As the science of quality improvement in health care advances, the importance of sharing its accomplishments through the published literature increases. Current reporting of improvement work in health care varies widely in both content and quality. It is against this backdrop that a group of stakeholders from a variety of disciplines has created the Standards for QUality Improvement Reporting Excellence, which we refer to as the SQUIRE publication guidelines or SQUIRE statement. The SQUIRE statement consists of a checklist of 19 items that authors need to consider when writing articles that describe formal studies of quality improvement. Most of the items in the checklist are common to all scientific reporting, but virtually all of them have been modified to reflect the unique nature of medical improvement work.

This “Explanation and Elaboration” document (E & E) is a companion to the SQUIRE statement. For each item in the SQUIRE guidelines the E & E document provides one or two examples from the published improvement literature, followed by an analysis of the ways in which the example expresses the intent of the guideline item. As with the E & E documents created to accompany other biomedical publication guidelines, the purpose of the SQUIRE E & E document is to assist authors along the path from completion of a quality improvement project to its publication. The SQUIRE statement itself, this E & E document, and additional information about reporting improvement work can be found at http://www.squire-statement.org.

Reporting guidelines have played an increasingly prominent part in the biomedical literature in the past decade as researchers, editors, reviewers and readers have made serious attempts to strengthen the knowledge base of medicine. Guidelines have been developed for reporting randomised controlled trials (CONSORT),^1^ ^2^ studies of diagnostic accuracy (STARD),^3^ epidemiological observational studies (STROBE),^4^ meta-analysis and systematic reviews of randomised controlled trials (QUOROM),^5^ meta-analysis and systematic review of observational studies (MOOSE),^6^ and for other designs and content areas. To date, it has been challenging to publish reports of quality improvement efforts, in part because improvement work involves goals and methods that differ fundamentally from projects that study disease biology. The design, types of interventions, data collection and analysis related to quality improvement projects do not fit easily under existing publication guidelines.

In 1999, the editors of *Quality in Health Care* (since renamed *Quality and Safety in Health Care*) published guidelines for quality improvement reports (QIR).^7^ These recommendations were offered as “a means of disseminating good practice” so that practitioners may have the “opportunity to learn from each other as the science of audit and quality improvement matures” (p 76). Since their publication, QIR have been used in over 50 published articles. While the QIR provide an excellent structure for brief reports of improvement work,^8^ a more detailed and comprehensive set of publication guidelines will be useful for larger and more complex improvement studies.

Quality improvement (QI) is fundamentally a process of change in human behaviour, and is driven largely by experiential learning. As such, the evolution and development of improvement interventions has much in common with changes in social policy and programmes. At the same time, the high stakes of clinical practice demand that we provide the strongest possible evidence on exactly how, and whether, improvement interventions work. This double-barrelled epistemology makes the study and reporting of work in QI extremely challenging, particularly for the many “frontline” healthcare professionals who are implementing improvement programmes outside the academic and publishing communities. Finally, it is possible that many journal editors, peer reviewers, funding agencies and other stakeholders will not be familiar with the methodologies for carrying out, studying and reporting QI projects.^9^

The lack of consensus-driven guidelines is undoubtedly one factor contributing to the variation in reporting about improvement work and to the variation in completeness and transparency of that reporting. That variation has led to calls for slowing the pace of improvement work, and for increased diligence in applying traditional scientific (that is, experimental) research methods in improvement studies.^10^ Others have taken just the opposite position, and have called for pushing forward with widespread, short-cycle improvement work, in an effort to develop local learning about what works, in what situations and for whom.^11^ In our view, this is not an “either/or” proposition; rather, both traditional research and improvement work share a passion for developing and implementing interventions that benefit patients and systems. Research and clinical care delivery will both benefit from more consistent, clear and accurate reporting of improvement work.

Improvement efforts focus primarily on making care better at unique local sites, rather than on generating new, generalisable scientific knowledge. In that respect, most improvement work (like most politics) is local. Despite its local focus, local improvement frequently generate important new generalisable knowledge about systems of care and about how best to change those systems. Whether improvement interventions are small or large, simple or complex, the Standards for QUality Improvement Reporting Excellence (SQUIRE) guidelines provide an explicit framework for sharing the knowledge acquired by examining those interventions closely, carefully, and in detail.

## DEVELOPMENT OF THE SQUIRE GUIDELINES AND THIS E & E DOCUMENT

The SQUIRE guidelines were refined through a systematic vetting process with input from an expert panel and through public feedback. Specific details are provided elsewhere.^12^ In brief, this process involved the initial publication of guidelines in September 2005^9^; a period of open, public feedback; a consensus conference in April 2007; and an asynchronous, email feedback loop leading to this current publication. The SQUIRE guidelines “are intended primarily to support publication of the strongest and most definitive evidence on quality improvement in the permanent peer reviewed journal literature”^9^ (p 321). In this E & E document, we use the current SQUIRE guideline checklist (table 1) and identify exemplary examples from existing literature to show how each guideline item can be addressed. This E & E document is designed to aid authors as they embark on an effort to share their work through publication using the SQUIRE guidelines.

**Table 1:** SQUIRE Guidelines (Standards for QUality Improvement Reporting Excellence) with numbered items and description of each item in the checklist

Text section; item number and name	Section or item description
Title and abstract	Did you provide clear and accurate information for finding, indexing, and scanning your paper?
1 Title	(a) Indicates the article concerns the improvement of quality (broadly defined to include the safety, effectiveness, patient-centredness, timeliness, efficiency and equity of care)
	(b) States the specific aim of the intervention
	(c) Specifies the study method used (for example, “A qualitative study,” or “A randomised cluster trial”)
2 Abstract	Summarises precisely all key information from various sections of the text using the abstract format of the intended publication
	
Introduction	Why did you start?
3 Background knowledge	Provides a brief, non-selective summary of current knowledge of the care problem being addressed, and characteristics of organisations in which it occurs
4 Local problem	Describes the nature and severity of the specific local problem or system dysfunction that was addressed
5 Intended improvement	(a) Describes the specific aim (changes/improvements in care processes and patient outcomes) of the proposed intervention
	(b) Specifies who (champions, supporters) and what (events, observations) triggered the decision to make changes, and why now (timing)
6 Study question	States precisely the primary improvement-related question and any secondary questions that the study of the intervention was designed to answer
	
Methods	What did you do?
7 Ethical issues	Describes ethical aspects of implementing and studying the improvement, such as privacy concerns, protection of participants’ physical wellbeing and potential author conflicts of interest, and how ethical concerns were addressed
8 Setting	Specifies how elements of the local care environment considered most likely to influence change/improvement in the involved site or sites were identified and characterised
9 Planning the intervention	(a) Describes the intervention and its component parts in sufficient detail that others could reproduce it
	(b) Indicates main factors that contributed to choice of the specific intervention (for example, analysis of causes of dysfunction; matching relevant improvement experience of others with the local situation)
	(c) Outlines initial plans for how the intervention was to be implemented—for example, what was to be done (initial steps; functions to be accomplished by those steps; how tests of change would be used to modify intervention) and by whom (intended roles, qualifications, and training of staff)
10 Planning the study of the intervention	(a) Outlines plans for assessing how well the intervention was implemented (dose or intensity of exposure)
	(b) Describes mechanisms by which intervention components were expected to cause changes, and plans for testing whether those mechanisms were effective
	(c) Identifies the study design (for example, observational, quasi-experimental, experimental) chosen for measuring impact of the intervention on primary and secondary outcomes, if applicable
	(d) Explains plans for implementing essential aspects of the chosen study design, as described in publication guidelines for specific designs, if applicable (see, for example, www.equator-network.org)
	(e) Describes aspects of the study design that specifically concerned internal validity (integrity of the data) and external validity (generalisability)
11 Methods of evaluation	(a) Describes instruments and procedures (qualitative, quantitative or mixed) used to assess (a) the effectiveness of implementation, (b) the contributions of intervention components and context factors to effectiveness of the intervention and (c) primary and secondary outcomes
	(b) Reports efforts to validate and test reliability of assessment instruments
	(c) Explains methods used to assure data quality and adequacy (for example, blinding; repeating measurements and data extraction; training in data collection; collection of sufficient baseline measurements)
12 Analysis	(a) Provides details of qualitative and quantitative (statistical) methods used to draw inferences from the data
	(b) Aligns unit of analysis with level at which the intervention was implemented, if applicable
	(c) Specifies degree of variability expected in implementation, change expected in primary outcome (effect size) and ability of study design (including size) to detect such effects
	(d) Describes analytical methods used to demonstrate effects of time as a variable (for example, statistical process control)
	
Results	What did you find?
13 Outcomes	(a) Nature of setting and improvement intervention
	(i) Characterises relevant elements of setting or settings (for example, geography, physical resources, organisational culture, history of change efforts) and structures and patterns of care (for example, staffing, leadership) that provided context for the intervention
	(ii) Explains the actual course of the intervention (for example, sequence of steps, events or phases; type and number of participants at key points), preferably using a time-line diagram or flow chart
	(iii) Documents degree of success in implementing intervention components
	(iv) Describes how and why the initial plan evolved, and the most important lessons learned from that evolution, particularly the effects of internal feedback from tests of change (reflexiveness)
	(b) Changes in processes of care and patient outcomes associated with the intervention
	(i) Presents data on changes observed in the care delivery process
	(ii) Presents data on changes observed in measures of patient outcome (for example, morbidity, mortality, function, patient/staff satisfaction, service utilisation, cost, care disparities)
	(iii) Considers benefits, harms, unexpected results, problems, failures
	(iv) Presents evidence regarding the strength of association between observed changes/improvements and intervention components/context factors
	(v) Includes summary of missing data for intervention and outcomes
	
Discussion	What do the findings mean?
14 Summary	(a) Summarises the most important successes and difficulties in implementing intervention components, and main changes observed in care delivery and clinical outcomes
	(b) Highlights the study’s particular strengths
15 Relation to other evidence	Compares and contrasts study results with relevant findings of others, drawing on broad review of the literature; use of a summary table may be helpful in building on existing evidence
16 Limitations	(a) Considers possible sources of confounding, bias or imprecision in design, measurement, and analysis that might have affected study outcomes (internal validity)
	(b) Explores factors that could affect generalisability (external validity)—for example, representativeness of participants; effectiveness of implementation; dose-response effects; features of local care setting
	(c) Addresses likelihood that observed gains may weaken over time, and describes plans, if any, for monitoring and maintaining improvement; explicitly states if such planning was not done
	(d) Reviews efforts made to minimise and adjust for study limitations
	(e) Assesses the effect of study limitations on interpretation and application of results
17 Interpretation	(a) Explores possible reasons for differences between observed and expected outcomes
	(b) Draws inferences consistent with the strength of the data about causal mechanisms and size of observed changes, paying particular attention to components of the intervention and context factors that helped determine the intervention’s effectiveness (or lack thereof) and types of settings in which this intervention is most likely to be effective
	(c) Suggests steps that might be modified to improve future performance
	(d) Reviews issues of opportunity cost and actual financial cost of the intervention
18 Conclusions	(a) Considers overall practical usefulness of the intervention
	(b) Suggests implications of this report for further studies of improvement interventions
	
Other information	Were there other factors relevant to the conduct and interpretation of the study?
19 Funding	Describes funding sources, if any, and role of funding organisation in design, implementation, interpretation and publication of study

We formed an author group for the E & E document by inviting as participants people whose published work concentrated on specific areas of improvement and assigned a SQUIRE guideline item (table 1) to each author. The author identified one or more articles that exemplified the components of that item. He or she then wrote a commentary expanding on the item’s content and linking the example article to the contents of the guideline item. A few of the articles chosen as examples in this paper were written using the initial SQUIRE guidelines,^9^ but most were not. This E & E document demonstrates how it is possible to clearly write about improvement work using the SQUIRE guidelines. Also, many E & E sections include descriptions of what could have been added to enhance the example. Some may also link the contents of one section to another (for example, Study questions (item 6) and Summary (item 14)).

## USING THE E & E DOCUMENT

The examples encompass a range of study designs. Some examples relate improvement efforts at single institutions, some are multicentre trials and one reports on simulation activities to plan an intervention. In choosing these examples we made an effort to reflect the great heterogeneity that currently characterises the reporting of QI work. Use this E & E document as a reference to assist with your writing. If you are a novice author writing about improvement using the SQUIRE guidelines, we recommend reading this entire article to become familiar with all of the sections and the interactions and associations between the sections. If you are a veteran writer of manuscripts, perhaps you will scan this paper and delve deeper into some of the aspects of SQUIRE that are unique from other manuscript writing such as Intended improvement (item 5), the description of the Setting (item 8), or the Outcomes regarding the evolution of the intervention (item 13(a)). This E & E document is not primarily intended to guide your choice of methods, design or analysis for conducting improvement work, but rather as a guide for writing about improvement; however, we recognise that many of the elements included may be useful during the planning and execution of an improvement project. Some of the sections of this E & E contain more information about design than others. It may be helpful to use the SQUIRE guidelines themselves and this E & E to write an article “shell” as the project is unfolding. This could ease the final preparation of the manuscript for submission.

The SQUIRE guidelines are not exclusive of other guidelines. For example, an improvement project or effectiveness study that used a randomised controlled trial design should seriously consider using both the CONSORT^1^ ^2^ and the SQUIRE guidelines. Likewise, an improvement project that uses extensive observational or qualitative techniques should consider the STROBE^4^ guidelines along with the SQUIRE guidelines. We strongly believe that the SQUIRE guidelines are synergistic with other publication guidelines, and together these will produce a more complete manuscript than either set of guidelines alone.

We recognise that although the SQUIRE checklist contains 19 distinct sections, authors (or journal editors) may wish to combine information from two or more sections depending on the flow of information or the specific journal requirements. We recommend using these guidelines to describe the project clearly, not to strictly adhere to each guideline item. We caution against using the guidelines too rigidly. A test of the strict application of the Standards of Reporting Trials (SORT) guidelines to a manuscript in 1995 produced a paper that was less readable, illogically organised and lengthy.^13^ ^14^ Because many of the guideline items contain a large amount of information, strictly adhering to them would produce a manuscript that is probably far too long for many journal requirement (that is, usually about 3000 words). Some items can be summarised as a table (item 13(a), Outcomes), figure (item 12 Analysis and item 13(b), Outcomes), list or flow diagram (item 4, Local problem or item 8, Setting). We encourage authors to be cognizant of combining sections in order to keep manuscripts concise and clear. Although we believe that each of these items should be addressed, each item need not be a separate heading or subheading. These guidelines are best used as a way of checking the completeness, accuracy and transparency of drafts.

## ITEMS IN THE CHECKLIST

In this section, we provide a published example of each of the 19 SQUIRE checklist items (table 1). Examples are excerpts from articles printed in peer-reviewed journals. Each example is then followed by an explanation of the passage which comments on the content of the example. Some explanations provide additional guidance that may have strengthened the example in light of the SQUIRE recommendations.

### 1 Title

- Indicates the article concerns the improvement of quality (broadly defined to include the safety, effectiveness, patient-centeredness, timeliness, efficiency and equity of care)
- States the specific aim of the intervention
- Specifies the study method used (for example, “A qualitative study,” or “A randomised cluster trial”).

#### Examples

(a) Outcomes of a quality improvement project integrating mental health into primary care^15^

(b) Improving newborn preventive services at the birth hospitalisation: a collaborative, hospital-based quality improvement project.^16^

#### Explanation

The title of a quality improvement or patient safety paper should indicate that its primary focus is quality improvement or patient safety. The literature on quality improvement and patient safety also includes papers that are primarily theoretical and some that are large-scale studies about improving quality, so it is helpful for the title to indicate that the paper is a report of a specific intervention. The titles cited above refer to a “quality improvement project” indicating that it is report of a specific intervention. Including the words “quality”, “quality improvement” or “patient safety” is important for readers to identify the content and for the National Library of Medicine’s Medline database to apply the correct keyword classification in the medical subject headings (MeSH) for the article. Current MeSH headings include healthcare quality, access and evaluation; quality assurance, health care; quality control; quality indicators, health care; quality of health care; and total quality management.

The title may also describe the aim of the intervention and, if possible, give some indication of the type of setting and approach. Each of the above examples does this very well. While the title cannot convey all the information about the article, it will provide the first exposure of the material to the readers. A concise and complete title entices readers to continue reading into the body of the article.

### 2 Abstract

- Summarises precisely all key information from various sections of the text using the abstract format of the intended publication.

#### Examples

(a) **Background:** Inadequate blood pressure control is a persistent gap in quality care.

**Objective:** To evaluate provider and patient interventions to improve blood pressure control.

**Design:** Cluster randomized, controlled trial.

**Setting:** 2 hospital-based and 8 community-based clinics in the Veterans Affairs Tennessee Valley Healthcare System.

**Patients:** 1341 veterans with essential hypertension cared for by 182 providers. Eligible patients had 2 or more blood pressure measurements greater than 140/90 mm Hg in a 6-month period and were taking a single antihypertensive agent.

**Intervention:** Providers who cared for eligible patients were randomly assigned to receive an e-mail with a Web-based link to the Seventh Report of the Joint National Committee on the Prevention, Detection, Evaluation and Treatment of High Blood Pressure (JNC 7) guidelines (provider education); provider education and a patient-specific hypertension computerized alert (provider education and alert); or provider education, hypertension alert, and patient education, in which patients were sent a letter advocating drug adherence, lifestyle modification, and conversations with providers (patient education).

**Measurements:** Proportion of patients with a systolic blood pressure less than 140 mm Hg at 6 months; intensification of antihypertensive medication.

**Results:** Mean baseline blood pressure was 157/83 mm Hg with no differences between groups (P _ 0.105). Six-month follow-up data were available for 975 patients (73%). Patients of providers who were randomly assigned to the patient education group had better blood pressure control (138/75 mm Hg) than those in the provider education and alert or provider education alone groups (146/76 mm Hg and 145/78 mm Hg, respectively). More patients in the patient education group had a systolic blood pressure of 140 mm Hg or less compared with those in the provider education or provider education and alert groups (adjusted relative risk for the patient education group compared with the provider education alone group, 1.31 [95% CI, 1.06 to 1.62]; P _ 0.012).

**Limitations:** Follow-up blood pressure measurements were missing for 27% of study patients. The study could not detect a mechanism by which patient education improved blood pressure control.

**Conclusions:** A multifactorial intervention including patient education improved blood pressure control compared with provider education alone.”^17^

(b) “Background: Performing a lumbar puncture in an unwell child can cause anxiety in both the parent and the junior doctor. There is increasing evidence of post-lumbar-puncture complications in this age group.

Aims: To improve the documentation, consent for and technical performance of paediatric lumbar punctures to 100% of the required standard within 3 months.

Setting: The paediatric emergency department of a the Royal North Shore Hospital (University of Sydney, Sydney, Australia).

Participants: Paediatric emergency staff, including residents, registrars and consultants.

Methods: Medical records of 40 consecutive children who had undergone a lumbar puncture in the 6 months before the introduction of the lumbar-puncture proforma were reviewed. After introduction of the proforma, the records of 25 consecutive patients were reviewed to assess changes in the outcome measures. Before introduction of the proforma, junior medical staff were instructed in the procedure using specialised lumbar puncture manikins (Baby Stap; Laerdel, USA).

Results: Before introduction of the proforma, the median number of documented indicators was 4, out of a maximum of 12. There was almost no documentation of parental consent, patient complications and analgesia. Introduction of the proforma resulted in a highly marked increase to a median of 12 documented indicators per patient (p<0.01, 95% confidence interval 6 to 8).

Conclusions: The introduction of a lumbar-puncture proforma and formal teaching sessions using a paediatric manikin led to a marked improvement in the documentation of paediatric lumbar-punctures. Lumbar-punctures can be performed only by accredited medical officers who have achieved competency on the lumbar-puncture teaching manikin.”^18^

#### Explanation

The purpose of an abstract is to summarise the essentials of the work that was done, so readers can decide whether it is relevant to their practice or research. Abstracts are the only information included in electronic search databases such as Medline. Moreover, many readers never venture beyond the abstract; in fact, readers often start reading the abstract by reading the “bottom line”—that is, the conclusions section, and read the rest only if that catches their interest. For these reasons, the abstract—particularly the conclusions section—must be clear, succinct and accurate. To assist readers in locating articles that are of interest to them, abstracts should contain keywords that allow librarians to accurately classify the work into searchable categories.

Different journals have different requirements, so the above examples illustrate how the critical elements can be effectively incorporated into the abstract. Despite format differences, both abstracts clearly indicate that the paper reports the results of a quality improvement project.^17^ ^18^ The aim and/or primary objective of the project is clearly stated as well as the existing quality gap. A brief description of the setting and participants is also provided. The methods section describes the initial strategy for improvement while the results describe the impact of the changes. The evolution of the intervention may be difficult to convey in the limited space of an abstract, but should at least be mentioned if possible. The conclusion summarises the project and describes the “generalisable” lessons learned. Both of these abstracts are clearly written, so the reader is able to evaluate the contents of the entire manuscript quickly and intelligently.

### 3 Background knowledge

- Provides a brief, non-selective summary of current knowledge of the care problem being addressed, and characteristics of organisations in which it occurs.

#### Example

“Several studies document that patients frequently show signs and symptoms of clinical instability before actual cardiac arrest. Schein *et al* found that 70% of patients showed evidence of respiratory deterioration within 8 hours of cardiac arrest. Other warning signs include hypotension, hypertension, tachycardia, tachypnea, and altered mental status. Despite documentation of the patient’s clinical deterioration and physician notification, interventions are frequently inappropriate or absent. The rapid response team concept has evolved as a means of extending critical care outside of the intensive care unit to intervene early and to prevent deterioration to cardiac arrest. We recently implemented a rapid response team at our hospital and this study evaluates our early experience with this approach… .”

“Saint Anthony Central Hospital is a busy community-based urban Level I trauma center in Denver, CO. There are over 2,500 trauma admissions annually and just over 600 of the admissions have an Injury Severity Score >16. Moreover, St. Anthony Central Hospital functions as a tertiary referral center for multiple other facilities within the Centura health hospital Network. Experienced, board certified trauma surgeons are available inhouse 24 hours a day, 7 days a week (24/7). Similarly, critical care physicians are also inhouse and available at all times.”

“Before March, 2005, patient clinical deterioration was managed by the inhouse physicians, both trauma and critical care, after notification by the nurse caring for the patient. During the year before March 2005, we developed a rapid response team to react to patient clinical deterioration outside of the intensive care unit; in effect, bringing critical care to the patient’s bedside.”^19^

#### Explanation

To understand a quality improvement intervention clearly, readers need to understand how the intervention relates to general knowledge of the care problem. This requires the authors to place their work within the context of issues that are known to impact the quality of care. *Context* means “to weave together”.^20^ The interweaving of the issues that stimulated the improvement idea and a variety of spatial, social, temporal and cultural factors within the local setting form the canvas upon which improvement is painted. The explanation of context should go beyond a description of physical setting. A general description of the organisation (types of patients served, staff providing care and care processes before introducing the intervention) will help readers to determine if findings from the study are likely to be transferable to their own care setting. In studies with multiple sites, a table can be a convenient way to summarise differences in context across sites. The table can specify the structures, processes, people and patterns of care that are unique to each site and assist the reader in interpreting results.

The above example places the improvement work within the context of a recognised need to provide earlier intervention to patients who are experiencing clinical deterioration; however, the authors also provide substantial details about their facility as a Level I trauma centre that is highly invested in critical care delivery. This description will capture the attention of readers from similar facilities, as the results may have implications for their own institutions. Conversely, this also allows readers whose context is very different to interpret the results appropriately.

Whereas controlled trials attempt to control the context to avoid selection bias, quality improvement studies often seek to describe and understand the context in which the delivery of care occurs. Pawson *et al* propose using form of inquiry known as “realist evaluation” to explore complex, multi-component programmes that are designed to change performance. The relevant questions in realist evaluation are: “WHAT is it about this kind of intervention that works, for WHOM, in what CIRCUMSTANCES, in what RESPECTS and WHY?”^21^ Answering these questions within a quality improvement report requires a thoughtful and thorough description of the background circumstances into which the change was introduced.

The description of the background knowledge and local context of care will be blended between checklist item 3 (Background knowledge) and item 4 (Local problem). Placing information into the *exact* category is less important than ensuring the background knowledge, local context and local problem are fully described.

### 4 Local problem

- Describes the nature and severity of the specific local problem or system dysfunction that was addressed.

#### Example

“A completion rate of 90% [for screening colonoscopy] is considered acceptable and since the start of our programme has been accepted by the UK endoscopy community. Median colonoscopy completion rates found in an audit in three regions in the United Kingdom were between 57% and 73%, depending on how completion is defined, although some institutions report adjusted completion rates of more than 90%. In the United States, crude completion rates of 95% have been reported in large series (such as one series of 3465 colonoscopies), suggesting that a 90% completion rate is achievable in routine practice. The impact of incomplete colonoscopies on the success of a proposed national colorectal screening programme has been highlighted. We were aware that our colonoscopy completion rate was low, and we wished to attain the suggested standard so that our patients would benefit by avoiding subsequent barium enema or missed lesions.”^22^

#### Explanation

The introduction to a quality improvement paper should explicitly describe the existing quality gap. To be as specific as possible, authors should describe the known standard or achievable best practice, and then provide evidence that the local practice is not meeting that standard. These authors specified that 90% colonoscopy completion rates are achievable, yet local completion rates only range from 57% to 73%.

Comparing and contrasting local, regional and national outcomes helps to frame this study against other outcomes. By comparing colonoscopy completion rates in the UK with published completion rates in the United States, the authors provide documentation that better performance is both quantifiable and achievable. In the absence of a known achievable standard of care, authors need to state their rationale for the level of performance they set as their goal.

Although the implications of the quality gap need not be described in detail, a brief summary of the individual patient, local system and (if applicable) national implications help to frame the study’s importance. In the above example, the authors point to documentation that suboptimal colonoscopy completion rates can hinder a national healthcare initiative by allowing missed diagnoses, thus increasing morbidity and mortality, cost and medicolegal risk. Further implications of the quality gap can be addressed in the discussion section (see guideline items 15, Relation to other evidence and item 18, Conclusions).

### 5 Intended improvement

- Describes the specific aim (changes/improvements in care processes and patient outcomes) of the proposed intervention
- Specifies who (champions, supporters) and what (events, observations) triggered the decision to make changes, and why now (timing).

#### Example

“For many years our busy MICU [medical intensive care unit] struggled with CR-BSIs [catheter-related bloodstream infections]. In 2002 the CR-BSI rate was 44% higher than the national median of 5.2 per 1000 catheter-days. Like many hospitals, we had historically focused on infection surveillance and staff education. However, we recognised that more innovative strategies were needed to tackle this ongoing complex problem. The impressive utility of CQI [continuous quality improvement] in other healthcare settings, coupled with successful reports from other high risk industries, encouraged us to consider a CQI approach… .”

“Our primary goal was to show that real time measurement of CVC [central venous catheter] care was feasible in the MICU. We anticipated these new process measurements would guide CQI efforts and thereby lead to a reduced CR-BSI rate. To increase staff appreciation of the link between process measures and clinical outcomes, we fed bundled data back to providers.”

“We assembled a voluntary interdisciplinary team with at least one MICU leader, infectious disease expert, front line staff member, and quality improvement expert. The team’s goal was to develop a system for measuring the process of CVC care in real time with the understanding that this information would guide future improvement activities aimed at reducing infections. The team compiled a list of risk factors by reviewing the published literature on CR-BSIs, and then classified these risk factors depending on whether they occurred during the insertion or daily maintenance. When deciding where to focus their initial efforts, the team selected CVC insertion as the starting point. Several issues guided this decision: (1) most CVCs in the MICU were inserted by trainees and there was a high likelihood of practice variability; (2) there was strong evidence linking certain insertion behaviors with CR-BSIs; and (3) CVC insertion was easily defined and amenable to improvement.”^23^

#### Explanation

A quality improvement paper should describe the events or information that lead to the need for making a change. Authors should include specific data or other organisational factors that are relevant to the intended improvement. The idea for improving care rarely occurs isolated from other events. These authors acknowledge that infection surveillance and staff education efforts had been in place in the specified institution but were insufficient to reduce the rate of catheter-related infections. By describing existing and previous work to improve care, authors place the current effort in relation to former initiatives.

The aim of the improvement effort should be clearly described. As in this example, the statement contains information about the specific goal to be achieved and how this will be measured. The example above indicates that the project is the first part of a feasibility study to understand whether real-time measurement of catheter-related infections is possible. A second goal of the intended improvement is to assess whether the use of real-time measures will lead to reduction in the rate of infection.

In addition, authors should describe which aspect(s) of care was the focus of the intended improvement. The authors above choose to focus on the insertion of the central venous catheter, and they clearly describe the rationale for this choice both in terms of evidence from the existing literature and from local factors related to their intensive care environment.

Finally, a description of the intended improvement should include the people who were involved in the effort. These authors note four different members of the team and highlight their different roles. Including the roles of individuals who participate in an improvement effort provides the reader with information related to what expertise was brought to the group. Improvement efforts are often initiated by leaders in a unit, clinic, hospital or health care organisation. If such institutional support or expectations exist for an intended improvement, stating these factors can assist the reader in understanding what other organisational factors may have facilitated the improvement effort.

### 6 Study question

- States precisely the primary improvement-related question and any secondary questions that the study of the intervention was designed to answer.

#### Example

“In this article, we describe a generally applicable computer simulation model to analyze access times for hospital services and to investigate the capacity needed to reduce these access times. We give both the analytical model and the simulation model, including the results following implementation in two outpatient departments of the AMC [Academic Medical Center]. The questions that guided our study were:

1.
  - To what extent are analytical models appropriate and when is simulation essential for more insight?
  - Is it possible to generalize the models and results to other departments?”^24^

#### Explanation

The study questions are a fundamental component of the introduction because they clarify the study of the intervention and note the relation that these questions have to the aim of the study. The study questions are in contrast to the improvement aim that would be stated in item 5, Intended improvement. The aim specifies the intended clinical (or system) change while the study questions focus on the mechanism(s) by which the intervention works and how effective the intervention is, or can be. In the example, the authors make this distinction by outlining the goal of the intervention, the performance gaps that motivated it, then outlining the study questions in terms of how and why the authors evaluated the intervention. The detail provided by the study questions also lend insight into the context issues that impact the study (for whom it works, under what circumstances and why), so the study questions should flow naturally from the description of the context.

Study questions provide a synopsis of the investigator’s thinking about the local quality gap. The questions provide an important link between the perceived causes of that gap and the methodology selected to close the gap. In the above example, the investigators hypothesised that a mismatch between supply and demand was causing lengthy waits for new patients. Their clearly defined study questions served to focus the improvement intervention which is described in detail in their methods section. Study questions can help introduce the data that were used, which may include a blend of process and outcome measures from both quantitative and qualitative methods. Additionally, these study questions address the generalisability of the intervention, which in this case refers to the applicability of their models to other clinics. These generalisable concepts are addressed further in the discussion section (see guideline items 16(b) Limitations and 17(b) Interpretation).

### 7 Ethical issues

- Describes ethical aspects of implementing and studying the improvement, such as privacy concerns, protection of participants’ physical wellbeing, potential author conflicts of interest and how ethical concerns were addressed.

#### Example

(a) “Residents [of the nursing home] who were not expected to live more than 30 days from the date of enrollment (as judged by their attending physician and nurse),those residents with a history of anaphylactic or serious allergic reaction to fluoroquinolones, or those residents with advance directives precluding transfer to hospital were excluded. The research protocol was approved by St Joseph’s Healthcare Hamilton Research Ethics Review Board. All participants or their designated surrogate decision makers gave informed consent.”^25^

“Study nurses made routine visits to the nursing home to assess resident eligibility, discuss the trial obtain informed consent, and enrolled residents.”^25^

(b) “[T]he purpose of our improvement project was to improve the consistency and reliability of the caesarean delivery process and to achieve emergency caesarean delivery response times of less than 30 minutes.”^26^

#### Explanation

A quality improvement publication should reflect and affirm that ethical standards have been addressed in the planning and implementation of the study. As efforts to enhance the quality of health care grow, there is a need to ensure that ethical practices are reflected in these efforts. The ethical principles of autonomy (do not deprive freedom), beneficence (act to benefit the patient, avoiding self-interest), non-maleficence (do not harm), justice (fairness and equitable care) and do your duty (adhering to one’s professional and organisational responsibilities) form a foundation for the delivery of health care. Those same principles should underpin the planning, implementation, and publishing of quality improvements activities.

Some of the specifically noted ethical standards for quality improvement activities are derived from the above principles and include the following: social or scientific value from the QI activity (responsible application of limited resources); scientifically valid methodology (avoiding inappropriate harm to subjects and waste of resources); fair participant selection to achieve an equitable distribution of burdens and benefits (justice); favourable risk-benefit, limiting risks and maximising benefits (non-maleficence, avoiding harm); respect for participants by respecting privacy and confidentially (respect for individual autonomy); informed consent when minimal-risk quality improvement activities is part of patient’s treatment (respect for autonomy); and independent review of the ethical conduct and accountability of the quality improvement (professional, organisational and public accountability).^27^ Another ethical standard related to professional responsibility and organisational accountability is avoiding any real or perceived conflicts of interest. These ethical standards form the foundation for ensuring the ethical quality of QI.

Example (a) above is from a formal, planned QI research study designed to produce generalisable results. The reader has a clear understanding that the study addressed ethical standards because the protection of the participants was noted in both the research design and consent process. The other ethics guidelines were addressed as part of the independent review process. Because this example meets the generally accepted interpretation of a research project as reflected in the Common Rule as a “systematic investigation, including research development, testing, and evaluation, designed to develop or contribute to generalisable knowledge,”^28^ the quality improvement activity must adhere to these requirements. The article indicates that the QI protocol was reviewed and approved by the St Joseph’s Healthcare Hamilton Research Ethics Review Board. This statement verifies that the protocol received independent review, suggesting that its protocol was appropriately designed to address a scientifically and socially important question, used a fair participant selection process, obtained adequate informed consent process and involved a suitable risk-benefit ratio.

The authors in example (a) provided further detail on the ethical conduct of the project by stating that study nurses discussed the trial and sought informed consent to enrol eligible patients who met the inclusion criteria. Those nursing home “residents who were not expected to live more than 30 days…or those residents with advance directives precluding transfer to hospital were excluded” from the study.^25^ The reported findings indicate that some nursing homes chose not to participate in the study. The authors also report that “consent could not be obtained from next of kin” from a total of 89 nursing residents or their surrogate and five refused to consent to the study.^25^ The refusal to participate in the research study by some patients or their surrogates suggests the scope of the shared decision process—fostering an ethically grounded valid consent and refusal process.

In QI, ethical standards must also be addressed in the planning and implementation of the activity because improvement activities carry the potential for risk to patients or misuse of organisational resources. In order to foster adherence to ethical guidelines, healthcare organisations should ensure that “ethical oversight of QI…becomes part of an enhanced accountability system for professional responsibility and the supervision and management of clinical care” (p 688).^27^ An example of non-research quality improvement providing “systematic, data-guided activities designed to bring about immediate improvements in the healthcare delivery in particular setting”^27^ is found in example (b).^26^ The article describes a carefully planned and implemented process to improve the consistency and reliability of the caesarean delivery process in a small rural hospital; however, the publication does not explicitly indicate whether an independent review occurred, including the ethical conduct of the effort. It would be reasonable to expect that the QI project coordinators (authors) indicate that previously noted^27^ QI ethical guidelines were addressed in the planning and implementation of the project. This is not meant to suggest that an institutional review board (IRB) review is necessary for such non-research QI activity; however, authors should indicate that the QI activities they are reporting adhered to appropriate ethical guidelines in their publications.

The ethical concerns regarding potential conflict of interest in QI efforts were significantly different in the two publications. In example (a) which is a QI research publication,^25^ the authors provide detailed disclosure information regarding the authors’ contribution to the publication, financial disclosures, funding support, role of sponsors and acknowledgments. The extensive disclosure at the end of article is likely to be the journal’s requirement, and it provides the necessary transparency regarding any potential conflict of interest to be considered by the reader. Example (b)^26^ provides only a brief acknowledgement that does not fully address the real, potential or lack of conflict of interest. Because QI activities often seek to improve delivery practices, outcomes, effectiveness and efficiency of healthcare services at a particular facility, QI investigators may encounter potential conflicts between obligations to individual patients and the general care provided by the facility or organisation. Therefore, QI publications should include disclosure of conflict of interest information.

The central issue in these examples is not whether the publication is a research project or not, rather that the reader is assured that ethical standards have been addressed in the planning and implementation of the QI activity. Transparency regarding the adherence to ethical standards is a vital aspect of the preparation of the manuscript and the journal’s decision to publish—reinforcing the point that ethics is an essential element of quality and of quality improvement activities.^29^

### 8 Setting

- Specifies how elements of the environment considered most likely to influence change/improvement in the involved site or sites were identified and characterised.

#### Example

“Alice Peck Day Memorial Hospital (APD) is a 32 bed hospital located in Lebanon, New Hampshire, USA. The birthing center has six labour/delivery/recovery/postpartum rooms and a Level I nursery. This unit is usually staffed by a minimum of two nurses. During delivery, one-on-one nursing is provided. When this project started in 2001, two independent physician groups, employing four providers, admitted obstetric patients. Paediatric services were provided by 16 physicians distributed among four independent practices. Three anaesthesia practitioners provided continuous coverage. Caesarean deliveries are performed in the hospital’s main operating room. The operative team consisted of the attending obstetrician, the first assistant, the paediatric provider, the anaesthesiologist, two operating room staff and the birthing centre nurse.

As the hospital has grown, physician groups have expanded, consolidated and changed practice patterns. Currently three obstetricians and three CNMs [clinical nurse midwives] are employed by the hospital. One additional CNM has a small independent practice. The majority of in-house paediatric services are now provided by one hospital-owned practice that employs four family practitioners and two paediatricians. Anaesthesia and operating room services are unchanged; although, we no longer consistently have a first assistant at all caesarean deliveries.”^26^

#### Explanation

This section describes the specific and relevant elements of the setting in which the improvement effort took place. It is an opportunity to expand upon the basic description that may be contained in the Background knowledge (item 3) and Local problem (item 4) items. In the example, this hospital is located in a rural part of New Hampshire, and “rural” is also included in the title of this article (item 1). Rural sites often are hospitals with smaller patient capacity (that is, number of hospital beds) and with smaller professional staff. The authors further describe the challenges of meeting emergency caesarean section time targets with the limited resources at this site, thus illustrating the financial and staffing limitations in this setting. Improvement in this example was not a matter of hiring a coordinator or purchasing new equipment; it was predicated on the current staff using the currently available resources, so describing the current situational factors—and how they changed throughout the study period—is stated. Implementing the changes described in this paper could be much different in a highly staffed, urban hospital with different staffing resources.

The paper later describes that the improvement efforts were undertaken in response to a sentinel event that involved a caesarean delivery response time, thus providing information regarding the underlying culture in which the change took place. In a small hospital, an adverse outcome affects almost everyone on staff. Describing how this event galvanised support for change was key in this article. This example could have been strengthened by more specific information about the degree of leadership support from middle and senior management as well as information about the history of previous change efforts, if any.

This framework of context and process description is necessary for readers to understand the generalisability (external validity) of the report. Identifying, understanding, and making changes to the processes and structures of care are essential to improvement work. This is in contrast to controlled trials where context is held constant through the design (for example, randomisation, blinding) and analysis (for example, regression analysis). Clearly describing the context for the reader will assist in their efforts to extrapolate to their own setting.

### 9 Planning the intervention

- Describes the intervention and its component parts in sufficient detail that others could reproduce it
- Indicates main factors that contributed to choice of the specific intervention (for example, analysis of causes of dysfunction; matching relevant improvement experience of others with the local situation)
- Outlines initial plans for how the intervention was to be implemented—for example, *what* was to be done (initial steps; functions to be accomplished by those steps; how tests of change would be used to modify intervention), and *by whom* (intended roles, qualifications and training of staff)

#### Example

“Twenty-three primary care organizations voluntarily enrolled in the Depression Breakthrough Series. This intervention was coordinated by the Improving Chronic Illness Care team and the Institute for Health Care Improvement and supported by the Robert Wood Johnson Foundation. Six private-sector organizations each paid a fee of $12,500, and 11 public-sector organizations that were financed by the Health Resources and Services Administration received a scholarship from the Robert Wood Johnson Foundation to cover the fee. Each team (typically a physician, a care manager, and a coordinator) attended three learning sessions for training by institute faculty on “plan-do-study-act” cycles and other techniques for rapid quality improvement and by experts in implementing the chronic care model. Between sessions, teams attempted to make changes and participated in monthly cross-site conference calls with expert faculty.

On the basis of the chronic care model, which envisions the delivery of effective treatment for chronic illness by restructuring care systems, teams were encouraged to design programs that implement changes within each of six areas… .”^30^

#### Explanation

Readers can best understand the meaning and applicability of a quality improvement intervention when the key features of the planning process are well documented. Unlike tightly controlled research studies, which tend to be studies of “conceptually neat and procedurally unambiguous” interventions like drugs, tests or procedures, studies of quality improvement interventions are intended to help others predict whether a particular approach to changing performance is likely to result in improved routine care. Routine care cannot be dissociated from its contexts, so readers need to understand how a quality improvement intervention influenced and was influenced by the context within which it was carried out. The planning of the intervention is a key component of this.

In the example, contextual factors include intervention financing and number and type of organisations. Additional contextual factors, such as the size, academic affiliations, rural or urban location, region of the country and characteristics of the patient population are known to have major effects on quality of care and intervention implementation. Organisational characteristics of participating sites, particularly those characteristics relating to elements of the chronic illness care model, as well as those describing an organisation’s culture or leadership, should also be included in this section.

The example contains very specific information about the details of the initial intervention. It specifies the organisations, teams, faculty members and theoretical background (PDSA, model for improvement, chronic care model) used in setting up the interventions. Basic planning steps included three learning sessions and monthly telephone calls. Local improvement teams modified and adapted their intervention plans at each step. All of these facets of the initial planning process help the reader to anticipate the conditions under which the quality improvement intervention’s results would be most applicable.

In some cases, intervention planning focuses on policy change, with little structuring of the specific change in the local setting. In other cases, an organisation, community or microsystem within an organisation may adopt a previously designed set of changes as its intervention. If the focus is adoption of pre-set changes, the planning phase may particularly emphasise the development of evaluation goals, standards and methods. In each case, the report of the planning phase should identify the focus of the quality improvement intervention and the design decisions related to that focus. For example, if the QI intervention is being developed de novo (or if it requires substantial adaptation from previous models), the report of the planning phase write-up should focus on piloting changes, such as through PDSA cycles. If the QI intervention is a policy change alone, the planning phase write-up should focus more on what was done to garner feedback from stakeholders or to pilot early evaluation designs.

In many cases, such as in the example above, an organisation or community intends either to substantially adapt a previous intervention. Describing the financial and technical support for the planning process and for the interventions is important, since both the type and degree of support provided for both the planning phase and the interventions can influence the scope of changes that are undertaken. In addition, as is usually the case, the specific sources of financial support may raise issues of actual or potential conflicts of interest, even when the sources are not for profit. Some of this information may be cross-referenced with information in the sections about Ethical issues (item 7) and/or Funding (item 19). Readers need to understand each of these issues to predict how well the quality improvement intervention may apply under other circumstances.

### 10 Planning the study of the intervention

- Outlines plans for assessing how well the intervention was implemented (dose or intensity of exposure)
- Describes mechanisms (theories) by which intervention components were expected to cause changes, and plans for testing whether those mechanisms were effective
- Identifies the study design (for example, observational, quasi-experimental, experimental) chosen for measuring impact of the intervention on primary and secondary outcomes, if applicable
- Explains plans for implementing essential aspects of the chosen study design, as described in publication guidelines for specific designs (see, for example, www.equator-network.org)
- Describes aspects of the study design that specifically concerned internal validity (integrity of the data) and external validity (generalisability).

#### Example

“The specific aim of this project was to eliminate catheter-related blood stream infections (CRBSI) throughout intensive care units (ICUs) in Michigan. We used a multiple time series design. The study population was all Michigan ICUs willing to participate.

The primary outcome (dependent) variable was the rate of CRBSI…Hospitals were to adhere to the Centers for Disease Control (CDC) definition of catheter-related bloodstream infection during the study period…The quarterly rate of infection was calculated as the number of infections per 1000 catheter-days for each 3-month period. Quarterly rates were assigned to one of eight categories on the basis of when the study intervention was implemented: at baseline, during the implementation period, or during one of six 3-month intervals occurring up to 18 months after implementation.

…To understand compliance with the interventions, teams completed a monthly form, called the team check up tool that evaluated what activities they did during the prior month, the number of times they met with their team and senior leader, and the barriers they were facing. All teams participated in bi weekly conference calls to discuss the project.”^31^

#### Explanation

This study highlights the multiple trade-offs that occur in designing and reporting a quality improvement study. This report states the design as a multiple time series in which all ICU sites willing to participate were included. While a cluster randomised design may have been more robust, it was not tenable because all the teams wanted the intervention and none wanted to be randomised to the control—a common desire when testing the implementation of known, effective interventions. Although it is not necessary to note this, alternative designs that were considered and deemed to be untenable should be noted in the limitations. The effectiveness of the intervention for eliminating catheter related blood-stream infections (CRBSI) was clearly known from previous studies,^32^ so this was an assessment of the effectiveness of the implementation of the interventions across many sites. The description of this should account for the size and scope of the implementation. This example was a large-scale implementation across a large geographical area, so the description includes mention of the study design (“multiple time series”), the operational definition of the main outcome measure (“the Centers for Disease Control (CDC) definition of catheter-related bloodstream infection”) and how data integrity was maintained (example not included). An improvement study at one location may include a very different, but equally precise, description of the plan of the study of the intervention.

There is often a struggle between collecting data that are scientifically sound yet feasible. Quality improvement studies often have limited resources and data collection may be voluntary. As such, this study notes the reduced quantity—but not quality—of data collected. The definition of CRBSI, the main outcome measure, was clear (although not included in the above example), but there were no data collected about secondary outcome variables such as the type of organism or adherence to the evidence-based recommendations. There was no valid and feasible way to do this as independent observation would have required placing an observer in the ICU for substantial amounts of time; a costly endeavour for central lines which are often placed at irregular intervals. The study also reported on data quality control including data definitions, training and amount of missing data. Nevertheless, because of insufficient resources, it did not report more robust methods of data quality control such as duplicate data entry or inter-rater reliability.

This study demonstrates how the intervention was guided by the study goal: eliminate CRBSI. When designing any study, the team invariably faces trade-offs. This example demonstrates how the aim, the design of the study and the measures were identified and implemented across multiple sites in a large geographical area. Improvement designs are sometimes limited by the systems willing to make changes. There are many ways to design a quality improvement study, and the study aims guide that design and the analysis. When writing about the study of the interventions, be sure to integrate the SQUIRE publication guidelines with guidelines for reporting on the specific study design whether it is a qualitative epidemiological study (STROBE),^4^ a randomised controlled trial (CONSORT)^1^ ^2^ or other design.

### 11 Methods of evaluation

- Describes instruments and procedures (qualitative, quantitative or mixed) used to assess (a) the effectiveness of implementation, (b) the contributions of intervention components and context factors to effectiveness of the intervention and (c) primary and secondary outcomes
- Reports efforts to validate and test reliability of assessment instruments
- Explains methods used to assure data quality and adequacy (for example, blinding; repeating measurements and data extraction; training in data collection; collection of sufficient baseline measurements).

#### Example

“All indicators were dichotomous variables thought to be amenable to simple quality improvement measures. In general, the quality indicators allow for a longer time frame to administer the clinical intervention (vaccine, aspect of physical examination, or laboratory test) than suggested by the ADA [American Diabetes Association] guidelines. This leniency means that performance for these indicators should be better than for the ADA guidelines because decreasing the time frame for the clinical intervention would probably decrease average indicator performance.

We ascertained from the medical record whether the following appropriate care was performed for each eligible patient at least once during the 18-month period: (1) measurement of long-term glucose control as reflected by at least 1 test of glycosylated hemoglobin (hemoglobin A_1c_) or fructosamine, (2) measurement of serum cholesterol, (3) measurement of serum triglycerides, (4) measurement of serum creatinine, (5) performance of in-office foot examination, and (6) administration of influenza vaccine. Performance on an indicator was quantified by dividing the number of eligible patients who received the item by the total number of eligible patients.

…All data for the quality measures were obtained from chart review according to methods previously described. Charts were photocopied and abstracted centrally using MedQuest, publicly available software developed for HCFA [Health Care Finance Association] (http://www.hcfa.gov). The ACQIP [Ambulatory Care Quality Improvement Program] investigators developed a standardized chart review protocol and refined the protocol through pilot testing. As part of the protocol, abstractors underwent intense training with competency certification. The MedQuest chart review module contained standard lists for variable synonyms, medications, diagnoses, and procedures. Throughout the chart abstraction period, 5% of charts were randomly sampled for dual abstraction and physicians evaluated chart abstractions for validity. Validity and reliability of all key variables were at least 95%.”^33^

#### Explanation

The description of the methods of evaluation outlines *what* the study used to quantify improvement, *why* the measures were chosen and *how* the investigators obtained the data. The measures chosen may be outcomes or process measures, continuous or categorical, biological or behavioural. The measures also must be sensitive enough to detect meaningful change in the processes and outcomes. Measures should have an accepted, clear operational definition so that changes in the values can be determined statistically and, more importantly, significant to the clinical problem being studied. Different perspectives, such as provider, patient, payer or societal, should also be considered during the delineation of measures.

In the example, the improvement project focused on achievable benchmarks in diabetes care. The study’s measures were the proportion of the population receiving certain tests or interventions. These are process measures. If the study had looked at the change in haemoglobin A_1c_ values, the number of influenza cases prevented or change in the quality of life of the patients, then the measures would have been outcome measures.

Whether process or outcome, measures should reflect a reasonable range of potential changes that may result from the intervention. Haemoglobin A_1c_ is frequently used as an outcome measure to determine adequacy of glycaemic control in diabetes-related studies; however, both the disease and the change effort may cause or alleviate a burden to the patient or to the provider. Therefore, it may be appropriate to measure behavioural or functional factors such as quality of life or satisfaction. Cost is often an important variable in change efforts. It is imperative that the investigator look at the process and the proposed improvement plan and determine a reasonable, balanced approach (sometimes referred to as a “data dashboard”) to measure those factors that are likely to be affected by the improvement intervention. Consensus panel guidelines and quality indicators are helpful in guiding the investigator’s choice of measures, but the local environment and problem should be considered to optimise the measures chosen.

Once the measures have been chosen, the investigator needs to develop operational data definitions, collection forms and determine how the data will be collected. The methods of data collection and data quality management should be described in the paper so that others may replicate the project. In the example above, chart abstraction was used to collect the data. The authors explain in detail how the data were abstracted and how a random sample was chosen for dual abstraction to confirm the adequacy of the abstraction method and the data.

The manuscript should also ensure that the factors being study are reliably measured. For biological specimens, this may include describing the laboratory process used or the method of specimen collection and handling. Behavioural domains should be measured with validated instruments. If a new scale is created for the change effort, then the reliability and validity of the scale should be described. The validity should include a construct validity model or comparison to a gold standard, if available. This informs the reviewer and reader that the measure accurately represents the factor or domain that was being assessed.

### 12 Analysis

- Provides details of qualitative and quantitative (statistical) methods used to draw inferences from the data
- Aligns unit of analysis with level at which the intervention was applied (if applicable)
- Specifies degree of variability expected in implementation, change expected in primary outcome (effect size) and ability of study design (including size) to detect such effects
- Describes analytical methods used to demonstrate effects of time as a variable (for example, statistical process control).

#### Examples

**Figure 1:**
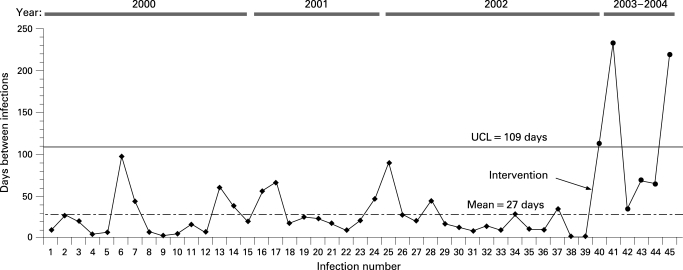
Example of a statistical process control chart (g-chart) with notation of when intervention occurred. UCL, upper control limit.

(a) “By monitoring the number of days between infections, the g chart has greater detection power for rare events compared with conventional binomial based approaches. Whenever a CR-BSI [catheter related bloodstream infection] occurred we added the data point to our g chart and calculated the number of days from the previous infection. …The g chart is a type of statistical process control (SPC) chart and therefore requires a basic understanding of the principles inherent to SPC. … At the start of the project we constructed a baseline (preintervention) g chart by querying the NNIS [National Nosocomial Infection Surveillance System] database from 1 January 2000 to 31 October 2002 (fig 1, observations 1–39). We only plotted CR-BSIs on the g chart for catheters inserted by the MICU - for example, dialysis catheter CR-BSIs were not included on the g chart.

“We measured the average time between infections (27 days) and used Benneyan’s method for calculating an upper control limit (UCL = 109 days) at three standard deviations above the pre-intervention mean. During the pre-intervention period the number of days between infections was consistently below the UCL. The absence of any points above the UCL suggested that the variation in time between CR-BSIs was inherent to our current process of care (that is, common cause), and that the proper way to reduce the CR-BSI rate was through process redesign or CQI. Our goal was to reduce the CR-BSI rate and thereby increase the time between events. Based on the g-chart, we considered our intervention successful if data points fell above the UCL since this would correspond to a decreased CR-BSI rate.”^23^

(b) “All audiotaped interviews were transcribed verbatim. Two researchers...independently reviewed the manuscripts and marked comments about barriers to adherence. Remarks of professionals were compared and classified into categories of potential barriers to physician adherence according to a conceptual model…. The two reviewers discussed all the remarks that they had individually highlighted and classified until consensus was reached. They consulted a third researcher to make a formal judgment about differences in classification. If controversy remained, the comment was considered ambiguous and was excluded.”^34^

#### Explanation

The analysis plan is intimately related to the study design. A defining characteristic of quality improvement is to show that the strategy for change (which is often multi-faceted) works to bring about a measurable difference in the process or outcome measures. Such demonstrations have resulted in a plethora of before-after studies comparing two data points, one value representing the pre-intervention period and the second value representing the post-intervention period. Such two-point before-after analyses are weak demonstrations of change; consequentially, they are generally considered pre-experimental, and hence acceptable as secondary, hypothesis-generating contributions, which require additional hypothesis-testing analysis.

Strong demonstrations of improvement projects overcome the limitation of before-after analysis by capitalising on the concept of replication. The strength of replication is that it accumulates confidence that the intervention produces the pattern of change observed in the results. Example (a) from Wall *et al* uses replication in the form of statistical process control (SPC) analysis to demonstrate a change from pre-intervention (baseline phase) to post-intervention (implementation phase). The authors state the type of SPC that was used (“g-chart”), the timeframe for the baseline data (“1 January 2000 to 31 October 2002”) and the inclusion criteria for the data (“for catheters inserted by the MICU”), thus specifying the analysis for the main outcome measure in the study. SPC is within the family of time-series analyses that plot multiple points, and where each point represents the operationally defined unit of measurement (such as a daily, weekly or monthly proportion, mean or time between events). We recommend consultation with a statistician familiar with time series analysis at the start of a study to ensure that the analysis is appropriate for the questions that are posed.

SPC requires a stable baseline from which to evaluate changes (improvements) that occur, but healthcare systems are also subject to secular trends that may appear in any given period of pre-intervention and during the intervention period. For these instances, other analytical techniques may be more appropriate. For example, the interrupted time series technique estimates the intervention’s effects and tests for statistical significance of change even with background noise from secular trends.^35^ ^36^ Alternatively, some authors may choose to use sophisticated statistical techniques that have been employed in longitudinal or time-dependent studies.^31^ These are appropriate for use in analysis of quality improvement, but these more sophisticated statistical techniques usually require consultation with a statistician for appropriate design, analysis and interpretation.

Also note that replication is a two-edged sword in the evaluation of complex social interventions. Particular interventions work well in some contexts but poorly, or not at all, in others. In the aggregate, therefore, introducing a complex social intervention into many different contexts is likely to lead to a null result overall, even when the intervention is effective in at least some settings.

Finally, not all quality improvement research is quantitative. Example (b) by Schouten *et al* demonstrates a brief description of qualitative analytic methods.^34^ Qualitative research is rich in discovering knowledge of root causes, understanding flow of process, formulating concept classifications and themes and gaining insight into mechanisms of change, context, perspective and perception. While simply relating anecdotes from qualitative studies can be powerful in affecting memory, attitudes and beliefs, qualitative research involves the use of systematic, rigorous and robust study techniques. Schouten’s example clearly describes how the interview data were transcribed, extracted, summarised and adjudicated. Other qualitative methods might use ethnographic or qualitative analysis software, provide evidence of inter-rater reliability or provide procedural detail for replicable data classification schemes. Authors who use qualitative study designs should also consult the guidelines for reporting of observational studies (STROBE)^4^ to be sure the methods and descriptions are consistent with those recommendations.

### 13 Outcomes

#### (a) Nature of setting and improvement intervention

- Characterises relevant elements of setting or settings (for example, geography, physical resources, organisational culture, history of change efforts), and structures and patterns of care (for example, staffing, leadership) that provided context for the intervention
- Explains the actual course of the intervention (for example, sequence of steps, events or phases; type and number of participants at key points) preferably using a time-line diagram or flow chart
- Documents degree of success in implementing intervention components
- Describes how and why the initial plan evolved, and the most important lessons learned from that evolution, particularly the effects of internal feedback from tests of change (reflexiveness).

##### Example

“The project began with the creation of four working groups that met regularly to work on specific component parts of the pathway. The results from each working group are summarized on Table 2 and discussed in detail below.”^26^

**Table 2:** Example of a display of the changes that occurred over time

	Summary of working group improvement efforts by phase of implementation				
Phase	Date complete	Interventions by working groups			
		Birthing centre nurses	Providers	Anaesthesia	Operating room
1	Summer 2002	Analysed tasks	Established dedicated CD phone line	Created OB anaesthesia questionnaire	Purchased dedicated OR infant “stabilet”
		Reassigned inappropriate tasks	Programmed on-call team members into computer	Placed completed questionnaire on birthing centre	Prepare OR for “STAT” CD each evening
		Eliminated unnecessary tasks (eg, transfer of patient to stretcher)	Programmed birthing centre phones with CD line on speed dial	Developed process for antenatal anaesthesia consults	Trained birthing centre nurses to check “stabilet” and OR preparedness each evening
		Organised supplies into CD kits	Instituted mightly test of system with mandatory call back requirement	Review OB high risk and VBAC lists each week	
2	Spring 2003	Replaced OR checklist with CD specific checklist	Designated codes to indicate “STAT” v routine CD	Recommend CBC, type and screen on all labouring women	
		Used mock CDs to identify steps in routine process that could be eliminated during emergency	Developed standardised terminology	Worked with providers to develop standardised terminology	Synchronised OR clocks with birthing centre clocks
		Distributed flow charts of “STAT” process	Created standardised brief operative note	Assisted with development of “STAT” process map	Assisted with development of “STAT” process map
		Finalised “STAT” process map	Assisted with development of “STAT” process map		
3	Spring 2004	Participated in OR skills day and “STAT” pathway mock drills	Participated in OR “skills day” and “STAT” pathway mock drills	Participated in OR “skills day” and “STAT” pathway mock drills	Participated in OR “skills day” and “STAT” pathway mock drills
		Empowered nurses and CNMs to transport patients to OR in emergency			
		Trained nurses in anaesthesia set up			
		Trained nurses in OR set up			

CD, caesarean delivery; OR, operating room; VBAC, vaginal birth after previous caesarean section.

##### Explanation

The results section should include a detailed explanation of the characteristics of the intervention, such as intensity and integrity, changes in the intervention over time, cost, attrition rates and sustainability. Because quality improvement studies evolve in “real-world” settings, and improvement involves context-sensitive change mechanisms, reporting the local context is essential (see item 4, Local problem, and item 8, Setting). If not included in the Local problem or Setting section, authors should include specific details about the clinic setting, patient population, previous experience with system change and how the context contributed to understanding the problem for which the study was designed. This article discussed these specifics in the Setting section (see item 8 above). Ideally, this section should also discuss the nature of the facility’s previous experience with, and commitment to, meaningful change.

It is likely that an intended improvement strategy will evolve over time in response to feedback from the environment and in response to changes in that environment over time. Improvement leaders and researchers should anticipate this evolution. When reporting on the results, this evolution should be captured. Often this is best done with a timeline or table that demonstrates changes over time, as in this example. The table from this example shows that the intended initial change strategy involved independent working groups that met in isolation. As the project progressed, however, it became clear that the work needed to be integrated. The table shows this evolution by indicating that by the spring of 2003, all of the working groups were participating in the same process of mock drills.

The success or failure of an individual improvement plan will be related to the inherent nature of the improvement as well as the interaction of that improvement within a particular setting. This example demonstrates the change in the improvement over time and how that affected the individuals in the organisation. Readers are then able to determine how their own organisation is similar to and different from the one whose change is described.

### 13 Outcomes

#### (b) Changes in processes of care and patient outcomes associated with the intervention

- Presents data on changes observed in the care delivery process
- Presents data on changes observed in measures of patient outcome (for example, morbidity, mortality, function, patient/staff satisfaction, service utilisation, cost, care disparities)
- Presents evidence regarding the strength of association between observed changes/improvements and intervention components/context factors
- Includes summary of missing data for intervention and outcomes.

##### Example

“Study Flow

…. Of the 205 providers, 23 were subsequently excluded after randomization because patients did not consent (n = 222) or because chart review showed that a patient was taking more than 1 medication (n = 264). …At trial completion, 975 patients (73%) had at least 1 follow-up blood pressure reading, including 255 of 324 (78.7%) in the provider education only group, 362 of 547 (66.2%) in the provider education and alert group, and 358 of 470 (76.2%) in the provider education, alert, and patient education group.

Outcome Measures: Systolic and Diastolic Blood Pressure

…the proportion achieving goal blood pressure {SBP ⩽140 mm Hg} differed in the 3 groups: 107 of 255 (42.0%) versus 148 of 362 (40.9%) versus 213 of 358 (59.5%) (P = 0.003) in the provider education; provider education and alert; and provider education, alert, and patient education groups, respectively. … There were no between-group differences in this secondary outcome {DBP<90 mm Hg} (P = 0.81) …

Process Measures: Intensification of Antihypertensive Regimen and Adherence

…[I]ntensification of antihypertensive medications was done in 32.4% of patients in the provider education only group, 28.5% in the provider education and alert group, and 29.1% in the provider education, alert, and patient education group (P = 0.81) … Medication adherence (pharmacy refills) was also measured after intervention, and there were no differences in medication adherence score among study groups … (P = 0.71).

… Death during Follow-up

During the study period…1.1% participants died (8 [2.5%] in the provider education only group, 3 [0.6%] in the provider education and alert group, and 4 [0.9%] in the provider education, alert, and patient education group; P = 0.027).”^17^

##### Explanation

**Figure 2:**
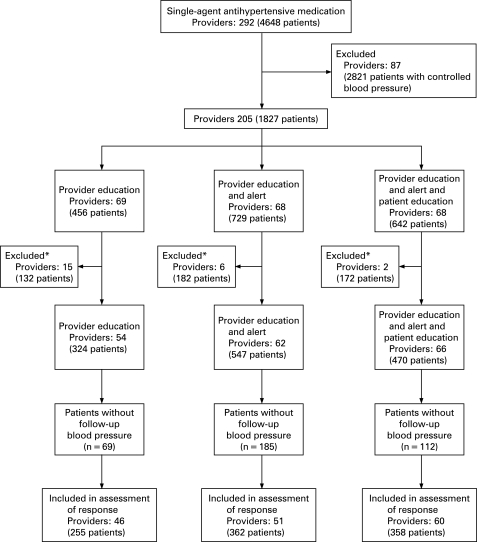
Study flow diagram.

In addition to providing specific details of the setting, intervention(s), and project evolution, it is equally important to describe changes in patient care and outcomes that occurred in response to the project. Data related to specific process and outcomes measures can be reported as shown in the above example. The study flow diagram (fig 2) combined with the text provides a clear picture of the study design at a glance and lay out the flow of participants over time.^37^ Providing this level of detail—in combination with a description of the evolution of the changes—helps readers to determine generalisability, or external validity, of the study.

Criteria to determine external validity for implementation studies have been suggested by Glasgow *et al* and include the following^38^: (1) representativeness of the sample (target audience, inclusion and exclusion criteria, participation, settings, individuals); (2) programme or policy implementation and adaptation (consistency, staff expertise, programme customisation); (3) outcomes for decision-making (significance, adverse consequences, programme intensity, costs, moderator effects—such as subgroups of participants/settings); and (4) maintenance and institutionalisation (long-term effects, sustainability, evolution, attrition). Each is demonstrated in the example. In the “Study flow” section of this example, the dropout rate and the magnitude of missing data over time are clearly stated. A situational analysis—which was not included in this examples—often includes the actual usage of the intervention, the degree of success in implementing the intervention and how and why the initial plan for improvement evolved (linked to the initial context description in item 4, Local problem, item 5, Intended improvement and item 8, Setting). Such analysis is sometimes considered beyond the scope of the main findings, but can be extremely important in understanding why an improvement intervention did or did not work in a particular setting. Finally, planned interventions that are not fully implemented as intended may result in less reliable findings, so these should also be reported in the manuscript.

Depending upon the study design, results may include means, proportions, standard deviations, risk ratios, confidence intervals or time-series. Furthermore, unexpected findings (harms or benefits), organisational impact, and difficulties implementing the intervention should be reported as well as the magnitude (effect size) and strength of associations. In the example, the indicated magnitude and strength of association with patient outcomes (Outcome measures: Systolic and diastolic blood pressure), processes of care (Process measures: Intensification of antihypertensive regimen and adherence) and other clinical outcomes (Death during follow-up) are all specified.

## 14 SUMMARY

- Summarises the most important successes and difficulties in implementing intervention components, and main changes observed in care delivery and clinical outcomes
- Highlights the study’s particular strengths.

### Example

“The new PSMMC [patient safety morbidity and mortality conference] provides a constructive venue for residents, fellows and faculty to express concerns about the healthcare system. Although altering the popular traditional MMC [morbidity and mortality conference] had inherent risk, the successfully executed transition has enhanced the overall experience for staff and faculty. The conference has remained a popular educational forum, with increased participation as measured by attendance. The growth in attendance noted seems to represent both higher levels of interest among medical residents as well as increased frequency of attendance by other healthcare professionals who are invited to participate in interprofessional care discussions. The response of the medicine residents and fellows has shown that they are capable and often enthusiastic about generating ideas for needed changes in systems of care. The attitudes of residents and fellows improved in key areas following the change, including the belief that positive departmental changes were likely to result from the analyses of medical errors and subsequent improvement actions.”^39^

### Explanation

In this example, the main improvements and outcomes are clearly summarised. The tension between the value of a brief summary of key findings versus a thorough restatement of all the findings in the study is demonstrated. The conscientious reader will digest the entire results and discussion sections for a more nuanced understanding of the outcome. This example was taken from a report of a single-institution study that employed multiple cycles of changes to an educational conference, and this summary flows logically from item 6, Study question, item 8, Setting, item 9, Planning the intervention, item 10, Planning the study of the intervention and item 13, Outcomes. The results section has tables and text to describe the details of the clinical and educational outcomes of this project. This level of detail is correctly placed in those sections and does not need to be repeated in the summary. The summary above focuses on the important strengths and outcomes (growth in attendance, attendance by non-physician healthcare professionals and increased resident physician attitudes), so authors must choose which are most important to share and which need to be left out.

### 15 Relation to other evidence

- Compares and contrasts study results with relevant findings of others, drawing on broad review of the literature; use of a summary table may be helpful in building on existing evidence.

#### Example

The self-management of asthma is recognised as an effective strategy in reducing morbidity. In the past, education programmes in the self-management of asthma have focused on primary schoolchildren or adults. Initiatives for asthma education for young people, aimed at individuals with asthma, have had minimal impact on asthma morbidity, and education programmes conducted in hospitals have problems attracting young people. In our study, the students who were educated by their peers had a lower number of reported asthma attacks and school absenteeism compared with the control group. Improvements in quality of life and asthma morbidity failed to cascade from year 10 into year 7 because the year 7 students only received the performances about asthma and not the peer-led teaching.

Interventions using peer education may have a higher chance of success in adolescence than other types of interventions. In a meta­analysis of 143 programmes in drug prevention in adolescents, the effect size was largest for peer teaching programmes than for other teaching strategies. Young people seem to prefer peers for advice, and change is more likely to occur if someone they can relate to or perceive as a role model relays the message. Additionally, peer educators enhance the programme’s effect by directing peer pressure in a positive direction.^40^

#### Explanation

Readers will want to know how the reported results relate to those of other published studies, both in terms of the results and impact of the intervention and in terms of the format and delivery of the intervention. Findings from individual studies are greatly enhanced when compared and contrasted with previously published articles. Authors should always compare their study results with appropriate findings from other relevant published work. This discussion should be as systematic as possible and not be limited to studies that support the results of the current study and, if available, there should be reference to existing systematic reviews. Use of tables and figures to help compare and summarise previous work may also be helpful.

Such information should help readers assess whether the results of the study are similar to those of other relevant studies, and whether the particular intervention that was used explains any noted differences. Reference may well be both to studies of the subject of the quality improvement research (such as asthma management in the above example) and to published literature on quality improvement interventions (for example, audit, academic detailing, change management strategies). The example provides a concise and reasonable assessment of recent literature by relating findings from the present study to those from other studies that are likely to be summarised in the cited meta-analysis. These comparisons provide the reader with a solid foundation upon which to anchor the findings of the current report. Finally, authors should explain how the new study adds to the existing body of evidence. This is not done in the example above, but is an important consideration.

### 16 Limitations

- Considers possible sources of confounding, bias, or imprecision in design, measurement and analysis that might have affected study outcomes (internal validity)
- Explores factors that could affect generalisability (external validity)—for example, representativeness of participants; effectiveness of implementation; dose-response effects; features of local care setting
- Addresses likelihood that observed gains may weaken over time, and describes plans, if any, for monitoring and maintaining improvement; explicitly states if such planning was not done
- Reviews efforts made to minimise and adjust for study limitations
- Assesses the effect of study limitations on interpretation and application of results.

#### Example

This study included multiple healthcare organisations and community agencies in a single metropolitan area rather than a single healthcare organisation and multiple community agencies from several regions. This approach minimised concerns of healthcare organisations in a single region about possible adverse selection of patients with dementia after the creation of enhanced services, and it more realistically reflected patterns of use of community resources encouraged by the chronic care model.

We adjusted for healthcare organisation provider but not for care manager because two healthcare organisations employed only one care manager each, thereby precluding our ability to distinguish healthcare organisation from care manager effects. We decided a priori to adjust for healthcare organisation rather than for care manager. The care managers received identical training and used the same assessments and treatment protocols, whereas we purposively recruited healthcare organisations with diverse characteristics and believed that healthcare organisation rather than care manager differences would influence outcomes. Dyads in the intervention group could be referred to multiple community agencies, depending on the service needs identified through the assessment of healthcare organisation care manager; referral to a community agency was a study outcome. Thus, we did not adjust for community agency care manager.

Our study sample was well educated, was predominantly white, had relatively few co-morbid conditions and was not institutionalised. Accordingly, the intervention may need to be modified for institutionalised patients and for those without a usual source of care and stable insurance. Secondary outcomes and some care process measures were self-reported, but multi-item scales met standards for reliability, and support for validity has been reported for several measures. As with almost any quality improvement intervention study, medical record abstractors could have discerned aspects of the study intervention, and we did not assess the extent to which abstractors were blinded to intervention status. It is possible that medical record documentation may incompletely reflect actual care processes, but we believe that observed differences reflect actual differences in care rather than differences in documentation because this care management intervention was based on a model in which multidisciplinary teams were engaged in delivering or facilitating the delivery of much of the recommended care.^41^

#### Explanation

The authors of this report identified a number of factors that could have introduced bias and affect the study measures. These included self-reporting of outcome and process measures, lack of blinding of medical record abstractors to the study interventions and the decision to adjust for healthcare providers but not for the community agency care managers. A description of efforts to control for these factors and minimise their effects either in the study design or the analysis of results should be included in the limitations section. In addition, insight into how the identified factors could have potentially affected the study measures, by either artefactually increasing or decreasing the effects of the described intervention, should be included in the limitations section.

This section should also explore why the particular study design was chosen and whether it reflects clinical practice in a realistic fashion. The authors in this example note that they chose their design with multiple healthcare agencies in a single area to reduce adverse selection of patients and to better reflect practice based on the chronic care model. Although this study design may be helpful for those who are planning care for multiple practices, this study design may not be as helpful for the individual practitioner who may be interested in implementing the described interventions into a single practice location.

This particular study was a randomised, controlled trial, a study design that typically makes use of multiple inclusion and exclusion criteria to ensure that the intervention and control groups are similar. Although this may improve the internal validity of the study by reducing the effects of confounding or selection bias from different study populations (or in this example, different clinical practices), it may also reduce the external validity or generalisability of the study as the included patient populations or practices may not resemble the diverse practices, providers or patients that exist in actual clinical care.

Finally, the limitations section should identify particular factors in the context and patient population that may affect generalisability. The success of improvement interventions depends on the specific contexts in which they occur; therefore, identification of unique traits or characteristics of the patient population, providers, institution or geographical setting is critical. A clear understanding of the local setting in which the intervention is implemented is important to the reader who is interested in applying the described interventions in their own particular setting and population of patients. The authors identified their patient population as unlikely to be representative of other populations across the country, suggesting that the study’s providers and practice settings are likely to be different from many other practices that care for patients who have dementia. The impact of this discrepancy could be mitigated by the inclusion of more information about particular aspects of the providers and practice settings that could affect the study outcomes.

### 17 Interpretation

- Explores possible reasons for differences between observed and expected outcomes
- Draws inferences consistent with the strength of the data about causal mechanisms and size of observed changes, paying particular attention to components of the intervention and context factors that helped determine the intervention’s effectiveness (or lack thereof) and types of settings in which this intervention is most likely to be effective
- Suggests steps that might be modified to improve future performance
- Reviews issues of opportunity cost and actual financial cost of the intervention.

#### Example

“…There are at least 2 plausible explanations or the significant reductions in hospital-wide mortality and code rate outside of the pediatric ICU [Intensive Care Unit] setting witnessed at our hospital vs. other pediatric hospitals. First, LPCH [Lucile Packard Children’s Hospital] serves a particularly high risk population of hospitalized children. …As a result, we speculate that LPCH has a higher proportion of children at risk for codes on its medical and surgical wards than do children’s hospitals with lower case mix indexes. …This difference in case mix index likely explains why this study found significant improvements in code rate and mortality rate only when including patients who met RRT [rapid response team] criteria, and why the study by Brilli *et al* found a significant decrease in code rate per 1000 patient-days only when using a single tailed statistical test. …A second possible explanation for our improved outcomes is that our postintervention period is substantially longer than both Melbourne’s (12 months) and Cincinnati’s (8 months).

“[T]hese improved outcomes have continued despite turnover among the resident physicians, nurses, and other staff, suggesting that the team rather than the education associated with the rollout is more likely the source of the improvements…. This explanation is supported by the decrease in RRT calls during the 19-month intervention period to levels below the effective threshold suggested by the Institute for Healthcare Improvement (20–25 calls per 1000 discharges).

“…Strategies other than an RRT that identify and respond to patients earlier in the course of a decompensation could have the same effect as an RRT. For example, 1 potential strategy to decrease codes outside of the ICU [intensive care unit] setting suggested by Winters *et al* is the integration of hospitalists. We are in a good position to evaluate a hospitalist intervention at a local level, because we introduced a medical-surgical ward hospitalist service 26 months (July 2003) before RRT implementation. Assuming that there was no delayed effect, the introduction of the hospitalist service, although valuable for multiple reasons, did not affect either mortality rate or code rate outside of the ICU setting….

“… Future research should focus on replicating these findings in other pediatric inpatient settings, including settings where children are treated in predominantly adult-focused hospitals, developing efficient methods for implementing RRTs, and evaluating the cost effectiveness of this intervention.

“…At LPCH, the RRT program was designed and implemented with no additional increase in funding for staffing, a decision supported by the time allocation required for calls during the first 19 months of the intervention.”^42^

#### Explanation

An improvement project is typically focused on identifying an association between an intervention(s) and some outcomes. The nature and degree of the change in outcome may arise from theory, research, or previous improvement studies and is usually stated in the Introduction section of the paper (item 5, Intended improvement and/or item 6, Study question). If the actual outcome deviates from the expected outcome or if the outcome is different from that observed in other published studies (as in the example above), the interpretation should include a discussion of the factors that prevented the expected outcome from occurring. In quality improvement studies, the explanation should include the situation factors (context) that may have led to these differences.

The interpretation section elaborates on the information reported earlier in the results section by describing the practical implications of findings. A statistical difference in outcomes is not sufficient as an explanation, so the internal and external implications of the findings should be explored. Competing theories and other causes of the outcomes should be investigated, such as the discussion regarding hospitalists in the example. Sources of bias and confounding are often addressed in the Limitations (item 16) section, but may be introduced here also. The intricacies about the various interventions and the contextual factors that may interact with the interventions in other settings could also be noted.

The interpretation often includes “lessons learned,” observations, and suggestions for improving the implementation of the interventions in other organisations. This may include the following:

- Background or cultural elements that should be in place before attempting implementation of the intervention
- Suggested sequence of steps for implementation
- Possible adaptations of the interventions
- Additional studies that could reinforce or enhance the findings.

The example includes a description of the possible future exploration regarding rapid response teams. This helps the reader identify where these current findings may and may not be applicable, thus enhancing the ability to interpret the context of the results.

Finally, it is often helpful to include some information that is relevant to the business case for the intervention that could help readers with the decision whether to adopt the change. One way to do this is to describe the resource and cost information associated with the reported study as in the above example. The authors also may describe the financial implications of the change in outcomes observed in the study. Authors should be aware of the intricacies and details that are required for an in depth economic analysis. The ISPOR RCT-CEA guidelines for cost-effectiveness studies that are alongside clinical trials may be helpful.^43^

### 18 Conclusions

- Considers overall practical usefulness of the intervention
- Suggests implications of this report for further studies of improvement interventions.

#### Example

“After demonstrating the feasibility of DMP [Discharge Medication Program] implementation throughout the 10 pilot institutions and its success in producing high adherence to discharge medication guidelines, IHC [Intermountain Health Care] expanded the program to include all 21 hospitals in the system. The DMP is now a solidly integrated program that continues to produce high rates of adherence to cardiovascular medication guidelines.

…Implementation of the DMP within the 10 hospitals required no additional employees. Adherence was tracked by using the existing hospital medical informatics infrastructure, adding only minimal cost to hospital operations. Consequently, we believe that cost was low relative to the numbers of lives potentially saved, especially when compared with recent improvements in cardiovascular care, such as drug-eluting coronary stents.

The comprehensiveness of the DMP is also substantially limited because patients were enrolled only if their principal diagnosis was cardiovascular. For instance, if a cardiovascular patient was admitted to the hospital with some other diagnosis, such as hip fracture, the DMP in our study would not have affected his or her discharge medications. Thus, this DMP could be used to treat even more patients if it were expanded to include everyone admitted with secondary diagnoses of cardiovascular disease.

Our study demonstrates that a relatively simple quality improvement project aimed at enhancing the use of appropriate discharge medications in patients hospitalized with a cardiovascular diagnosis is feasible and can be sustained on a large scale in a multihospital integrated system. Most important, our findings suggest that such a program may have substantial long-term lifesaving benefits across a broad system of integrated hospitals.”^44^

#### Explanation

The conclusion to a quality improvement paper should summarise the lessons learned from the project and detail the potential next steps for investigation. It should also describe how the successes of the project (if applicable) will be maintained or expanded. In the above example, the authors include the information that their improvement programme has been expanded to the other institutions in their hospital system and that improvement is being monitored through rates of adherence to cardiovascular medicine guidelines. This is one example of generalisability within the same organisation.

Conclusions may also describe how the local context might translate to other clinical arenas. Reflections as to what might be necessary to sustain the improvement or to test the improvement intervention in another care setting are helpful. Finally, suggestions as to possible next steps are also useful. The authors in this example suggest other patient populations who may benefit from the intervention. The goal of the conclusion is not only to summarise the local implications of the project, but also to provide insights to those readers who are considering the applicability of the presented intervention to their local care setting. Finally, it is useful for authors to consider, “What important questions does this study leave unanswered?” and “What studies would be most useful to answer those questions? Why?”

### 19 Funding

- Describes funding sources, if any, and role of funding organisation in design, implementation, interpretation, and publication of study.

#### Example

“Role of the Funding Source

This study was sponsored by the U.S. Department of Veterans Affairs (Veterans Integrated Service Network [VISN] Implementation grant), Clinical Research Center of Excellence, and Center for Patient Healthcare Behavior. The principal investigators and coinvestigators had full access to the data and were responsible for the study protocol, statistical analysis plan, study progress, analysis, study reporting, and the decision to publish the paper. The U.S. Department of Veterans Affairs–VISN 9 had the opportunity to comment on the manuscript before submission.”^17^

#### Explanation

Funding for improvement may arise from many sources: local institutional, a group of organisations that join together, a governmental granting agency, a foundation granting organisation or even from a third party payment. The description of the funding source can be brief, as in the example above, but should be thorough enough for the reader to understand which parties had a financial stake in the project. This example also clearly describes that the regional VA leadership in VISN 9 had an opportunity to comment on the paper. Transparency regarding the source of the funding as well as the level of input from the funding agency assists with the interpretation of the outcomes. This level of unambiguous clarity is particularly important in light of recent data that cast doubt on the role of authors in some studies funded from industry.^45^

**For more information about the SQUIRE guidelines and this E & E document, please visit http://www.squire-statement.org**

- Complete electronic version of this E & E document
- Hyperlink between E & E sections
- Provide comments and feedback about individual sections or the entire E & E
- Links to additional resources
- Updated news about the SQUIRE guidelines
- Contact SQUIRE authors with questions and comments

## COMMENT

Guidelines form an important bridge between the completion of a project and the sharing of the conclusions with others. As CONSORT^1^ ^2^ and STARD^3^ have provided guidance for reporting randomised controlled trials and tests of diagnostic accuracy, the SQUIRE guidelines provide structure for the reporting of improvement work. Because the guidelines themselves are brief by design, this E & E document provides additional specificity regarding details of that structure.

As you read and use these guidelines and the E & E document, you may have questions or comments about individual items in the guidelines, about examples that were chosen, about the guidelines or the E & E document as a whole. We intend this E & E as a starting point for ongoing dialogue about the implementation and evaluation of the SQUIRE guidelines. Please visit our website at www.squire-statement.org. The website has full and short versions of the guidelines, a glossary of terms used in the guidelines and this E & E document. The website is designed to facilitate a moderated, threaded discussion about individual guidelines and the guidelines as a whole. If you are submitting a manuscript to a journal that does not explicitly endorse the SQUIRE guidelines, we encourage you to refer the editor(s) and reviewers to the website so that they may become familiar with these guidelines.

The planning, implementation, analysis and reporting of improvement work continue to evolve. This E & E document provides a snapshot of existing examples that help to illustrate the many important and nuanced issues in doing, studying, and reporting QI work. Ideally, these examples will be supplanted by hundreds of additional ones in the coming months and years, as the SQUIRE guidelines are used widely throughout the improvement community.
